# Health Risks of Structural Firefighters from Exposure to Polycyclic Aromatic Hydrocarbons: A Systematic Review and Meta-Analysis

**DOI:** 10.3390/ijerph18084209

**Published:** 2021-04-15

**Authors:** Jooyeon Hwang, Chao Xu, Robert J. Agnew, Shari Clifton, Tara R. Malone

**Affiliations:** 1 Department of Occupational and Environmental Health, Hudson College of Public Health, University of Oklahoma Health Sciences Center, Oklahoma City, OK 73104, USA; 2 Department of Biostatistics and Epidemiology, Hudson College of Public Health, University of Oklahoma Health Sciences Center, Oklahoma City, OK 73104, USA; Chao-Xu@ouhsc.edu; 3 Fire Protection & Safety Engineering Technology Program, College of Engineering, Architecture and Technology, Oklahoma State University, Stillwater, OK 74078, USA; rob.agnew@okstate.edu; 4 Department of Health Sciences Library and Information Management, Graduate College, University of Oklahoma Health Sciences Center, Oklahoma City, OK 73104, USA; Shari-Clifton@ouhsc.edu (S.C.); Tara-Malone@ouhsc.edu (T.R.M.)

**Keywords:** firefighter, polycyclic aromatic hydrocarbons, occupational exposure, systematic review, meta-analysis

## Abstract

Firefighters have an elevated risk of cancer, which is suspected to be caused by occupational and environmental exposure to fire smoke. Among many substances from fire smoke contaminants, one potential source of toxic exposure is polycyclic aromatic hydrocarbons (PAH). The goal of this paper is to identify the association between PAH exposure levels and contributing risk factors to derive best estimates of the effects of exposure on structural firefighters’ working environment in fire. We surveyed four databases (Embase, Medline, Scopus, and Web of Science) for this systematic literature review. Generic inverse variance method for random effects meta-analysis was applied for two exposure routes—dermal and inhalation. In dermal, the neck showed the highest dermal exposure increased after the fire activity. In inhalation, the meta-regression confirmed statistically significant increases in PAH concentrations for longer durations. We also summarized the scientific knowledge on occupational exposures to PAH in fire suppression activities. More research into uncontrolled emergency fires is needed with regard to newer chemical classes of fire smoke retardant and occupational exposure pathways. Evidence-based PAH exposure assessments are critical for determining exposure–dose relationships in large epidemiological studies of occupational risk factors.

## 1. Introduction

The International Agency for Research on Cancer (IARC) has linked the chemicals detected in fires to cancer sites in humans [1]. Among the hundreds of substances from fire smoke contaminants, one potential source of toxic exposure is polycyclic aromatic hydrocarbons (PAH), which are ubiquitous byproducts of incomplete combustion processes [2]. PAH are the most commonly studied and best understood carcinogenic substances produced during firefighting activities [3,4,5]. Correspondingly, the European Human Biomonitoring Initiative (HBM4EU) has pinpointed nine prioritized chemical groups, including PAH, based on hazardous properties, exposure characteristics, regulatory status, public concerns, and technical feasibility [6]. Previous studies and meta-analyses have shown that all firefighters have an elevated risk of cancer. The National Institute for Occupational Safety and Health (NIOSH)-funded epidemiological studies of firefighters have reported excess cancer mortality (standardized mortality ratio (SMR) = 1.14, 95% CI 1.10–1.18) and incidence rates (SMR = 1.09, 95% CI 1.06–1.12) [7,8]. Likewise, a state-wide mortality study in Indiana found that the malignant cancer mortality rate for firefighters is higher than that for non-firefighters (odds ratio [OR] = 1.19, 95% CI 1.08–1.30) [9]. Career male firefighters in Florida had a significantly elevated risk of thyroid, skin, prostate, and testicular cancers (ranges of adjusted OR = 1.36–2.17, ranges of 95% CI: 1.27–2.66) [10]. According to several quantitative meta-analyses, the risk estimates for multiple myeloma, non-Hodgkin lymphoma, and prostate, colon, rectum, bladder, skin, and testicular cancer for firefighters are higher than those for non-firefighters [11,12,13,14]. Some of these cancer sites align with PAH carcinogenicity. The strongest evidence for the human carcinogenicity of PAH has been found for cancers of the colon, skin, lung, bladder, and kidney. In particular, benzo[a]pyrene, one specific compound in the PAH group, is classified by the IARC as a Group 1 human carcinogen, and is related to skin, lung, and bladder cancers.

Firefighters’ risk of exposure to PAH is likely to increase, as new compounds produce PAH when burned. In addition, structural firefighters are more likely to be exposed to fire smoke from modern synthetic materials. Although the number of fire incidents has decreased, the toxicity of chemicals encountered during fire suppression has increased. According to the National Fire Protection Association [15], U.S. fire departments responded to 1.3 million fires in 2018, which corresponds to one fire response every 24 s. Of these fires, 500,000 occurred in structures, which comprised 38% of total fires. Thus, in the U.S. alone, over 1.1 million firefighters may be exposed to PAH when they respond to a fire. Yet, the health impacts and economic losses due to carcinogenic exposure from PAH among firefighters have not been reported.

PAH exposure via inhalation is typical during fire suppression activities. However, the main exposure route for firefighters is not inhalation, as their self-contained breathing apparatus (SCBA) have a protection factor of 10,000 [16]. Rather, firefighters are covered with smoke-derived organic compounds, including PAH from fire-related activities and from their turnout gear [1,17,18,19,20,21,22]. While the inhalation of PAH from off-gassing after a fire activity or from accumulated PAH when donning or doffing personal protective equipment (PPE) cannot be ignored, transdermal absorption forms the major route of exposure, as recent studies have confirmed [23,24]. Thus, the concept and feasibility of two types of exposure routes as suggested in this systematic review and meta-analysis are validated by the prior literature on PAH. We systematically reviewed studies on the association between PAH exposure levels and contributing risk factors to derive best estimates of the effects of exposure on structural firefighters’ working environment in fire. These meta-results cannot be straightforwardly compared to those of previous evidence-based exposure assessments because the study settings vary widely with uncertain confounding factors.

## 2. Materials and Methods

### 2.1. Search Strategy

**Figure 1:**
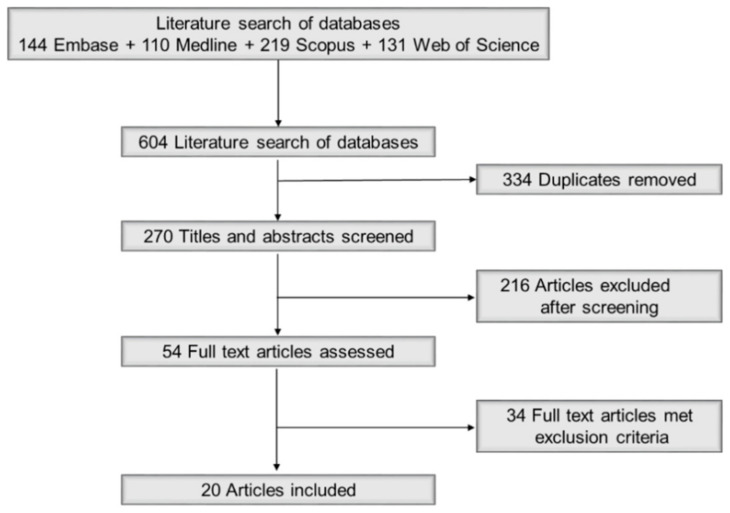
PRISMA flow chart of the literature screening and selection processes.

We followed the Preferred Reporting Items for Systematic Reviews and Meta-Analyses (PRISMA) guidelines to conduct our search of the literature [25]. To identify publications for this analysis, we searched the following databases: Embase (Classic + Embase OvidSP); MEDLINE (Epub Ahead of Print, in-Process and Other Non-Indexed Citations OvidSP); Scopus; and Web of Science Core Collection (Figure 1). We screened and searched for cited references using as the source items, and hand-searched journals containing relevant literature for additional articles. As the amount and quality of measurement data are limited, we did not include grey literature such as technical reports, including those from FEMA and NFPA, conference proceedings, or theses/dissertations. We customized our search strategy for each database by incorporating controlled vocabulary terms and/or keywords designed to retrieve literature relevant to the concepts ‘firefighters’ and ‘polycyclic aromatic hydrocarbons (PAHs)’. Searches were conducted in April 2020 and included literature published in any language. The detailed search strategies that reflect all terms and special features (e.g., limits, explode, focus, etc.) used for each database are listed in Supplementary File 1.

### 2.2. Screening and Selection Criteria

One investigator (JH) performed the initial screening of titles and abstracts against inclusion criteria. If the initial screening criteria were satisfied, the full text was retrieved. In the next screening, a second investigator (RA) cross-checked random half data points in the full text. A third investigator (CX) mediated when a consensus could not be reached, a safeguard that was not needed. The inclusion criteria were as follows: (1) structural firefighters involved in a fire activity, (2) quantitative assessment of PAH exposure levels, (3) inhalation or dermal exposure routes, and (4) original study. Exclusion criteria were as follows: (1) assessment of non-fire-related activity such as fire station or fire vehicle; (2) focus limited to biomarkers such as PAH-OH in urine; (3) laboratory-based experimental, epidemiological, or ecological study; (4) wildland or prescribed fires; (5) data reported as subset of a parent study; (6) PAH levels not reported; and (7) publication types, such as narrative or review papers, letters to the editor, book chapters, monographs, or commentaries. In addition, we excluded studies on the World Trade Center fire because the PAH exposure assessment was performed at least two weeks after the incident [26]. We also excluded studies examining the efficiency of laundering for the removal of residual exposure levels or of PPE decontamination which use interventional study designs, even if they focus on fire activities [27,28].

### 2.3. Data Extraction and Synthesis

The following data were extracted from the selected studies (Table 1): internal study ID, author, publication year, study location, exposure scenario (type of fire activity), study design, analytical methods and equipment, sample type, number of participants, number of burns, co-exposures, and fuel (burning materials). For the wipe samples, we focused on relative not absolute exposure values. Thus, a ratio between pre- and post-fire activity was predicted using a multivariable regression model. If no such information was available, we determined grouping based on the relevant exposure scenario for each study. For the air samples, we focused on the duration of sampling during the fire activity. When necessary, we extracted data points from bar charts, boxplots, and other figures. We standardized the units for air (µg/m^3^) and wipe (ng/cm^2^) to those used in most studies, showing the calculation when the original study reported the level of PAH using a different unit of measurement (see Supplementary File 2). Further detailed PAH analyte information was extracted and cross-checked from two publicly available databases: U.S. EPA CompTox Chemistry Dashboard (https://comptox.epa.gov/dashboard, accessed on 28 December 2020) and NIH PubChem (https://pubchem.ncbi.nlm.nih.gov, accessed on 28 December 2020). Williams et al. (2017) [29] and Kim et al. (2020) [30] described each database service and its utilization, respectively.

### 2.4. Statistical Analysis

We used the generic inverse variance method [31] for random effects meta-analysis, which was implemented by the R package meta and metafor. The missing values of arithmetic mean (AM), geometric mean (GM), and standard error (SE) were replaced using imputation method based on median and range. If a missing value could not be replaced, it was excluded from the meta-analysis. Two multi-regression models were explored for the moderator effect, such as the sampling time duration and time point, on the wipe and air samples, respectively, using the following exposure-related metrics: IARC classification, PAH structure, sampling location if wipe, time period for sample collection if air. The moderator effect was estimated with the SE and test *p*-value for the hypothesis of no effects. To mitigate publication bias, we visually inspected the funnel plot, which plots effect sizes against their standard errors or precisions. The heterogeneity was also investigated by I^2^ statistics and Q-test. Differences in study designs, methods of analysis, equipment and procedures, exposure scenarios, geographical and live training practices, and fuel materials could cause significant variation and heterogeneity in the overall effect estimates. Significance level was 0.05. All analyses reported here were conducted using the R 4.0.3 (R Core Team, Vienna, Austria).

## 3. Results

### 3.1. Literature Searches

**Figure 2:**
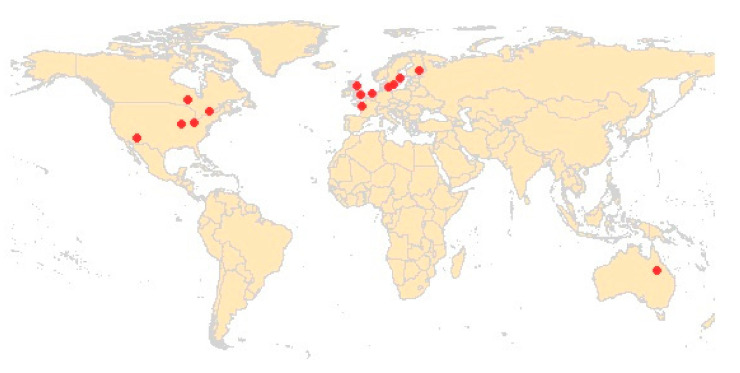
World map indicating where the selected studies were conducted.

Figure 1 provides a flow chart of the literature screening and selection process. Initially, 604 papers were retrieved from the four databases. After screening 270 papers and reviewing 54 relevant papers in depth, we identified 20 papers that were eligible for data extraction. The publication dates for the papers ranged from 1997 to 2020. All but four were published after 2014, which indicates that this field of study has only recently received attention. All studies were conducted in high-income countries [32], predominantly in North America and Europe, with two studies from a research group located in Australia (Table 1 and Figure 2). Studies in other countries may be limited due to a combination of a low priority placed on occupational and environmental exposure to fire smoke and a lack of appropriate equipment and facilities for conducting exposure assessments.

### 3.2. Description of Included Studies

Table 1 shows the characteristics of the selected studies. Most of the studies conducted the environmental exposure assessment in a controlled fire such as live fire training or a simulator, while only three studies were conducted during emergency fire suppression. Only one study assessed both live fire training and an emergency fire [36]. The sample size varied considerably, from 4 to 53 participants. Six studies used accumulated wipe sampling, five studies used ambient air sampling, and nine studies used both methods. Our systematic reviews for meta-analysis are not limited to either gaseous or particle-bound PAH, because not all of the studies we examined clearly distinguished between the gaseous/vapor-phase and the particle-phase PAH. We did report other co-environmental exposures measured along with PAH for comparing the studies. Other exposures that were measured shows a wide spectrum such as Volatile organic compounds (VOCs), metals, and particulate matters.

### 3.3. Meta-Regression of Skin Wipe Samples

PAH itself is characterized by a large and heterogeneous group of compounds. A variety of confounding variables were selected in the original studies, so, in order to control the results, we pooled the models with the highest level of adjustment. Considerable evidence was found to support two routes of exposure for structural firefighters—dermal and inhalation. This section describes the results for the dermal route.

In order to establish if dermal exposure increased after the fire activity, the differences between the mean PAH concentration for pre- and post-fire activity were estimated (Table 2). In this exposure-end point meta-analysis, we applied the concept of fold changes (FC), which was defined directly in terms of ratios in bioinformatics [50], to understand the magnitude of exposure changes. The FC represents a ratio of PAH mean between exposure (post) and non-exposure (pre) of the fire activities. The skin locations that had sufficient records were estimated using FC of the PAH mean. The FC varied from 0.49 to 2.0, with an FC greater than 1.0 indicating a higher PAH concentration post-fire activity. Except for three cases, all had an FC > 1.0, although no statistically significant differences were found between pre- and post-fire activity (*p*–values: 0.075–0.947). The three cases with FC < 1.0 may have been affected by the transfer of soot containing PAH from contaminated gear to the skin pre-fire activity [37].

In the original studies, the dermal samples were collected from various locations (back, collarbones, face, fingers, forehead, neck, and wrist). Of these, we analyzed three locations for which there was sufficient power to estimate FC. The neck had the highest FC overall, as well as pre- and post-fire activity. A plausible explanation for this pattern is that the neck is the only location in which the PPE hood provides minimal protection, with a double layer of porous flame-resistant fabric and, thus, no built-in vapor barrier [23,44]. Furthermore, sites with thinner skin, including the neck, tend to more quickly absorb PAH [23,42,44]. Our rationale for adapting the IARC classification is that the IARC Monograph program comprehensively evaluates the published scientific evidence on carcinogenic, including PAH, hazards to humans based on exposure-related data, cancer epidemiology in humans, carcinogenicity in experimental animals, and mechanistic and other related data [51]. Since the establishment of the IARC Monograph program in 1971, 121 agents have been classified as Group 1—carcinogenic to humans, 89 as Group 2A—probably carcinogenic, 318 as Group 2B—possibly carcinogenic, and 499 as Group 3—not classifiable. Each compound in PAH has been assigned to one of these four IARC classes. There were no analytes from group 2A (probably carcinogenic to humans). As group 1 (carcinogenic to humans) contains a single component of PAH, benzo[a]pyrene, a small number of records were compiled, which had high variability, up to SE = 2.74. Note that we also extracted the wipe samples of PPE but excluded them from the analysis due to the limited subset of records available.

Each original study selected a different number and components of PAH according to the available resources and the hypothesis. Of the various components of PAH, some studies analyzed up to 36 different analytes [46], while one study focused on a single analyte, benzo[a]pyrene, as a PAH marker [44]. Although there was a wide range of variability, naphthalene was the component mainly responsible for total PAH across the studies. Naphthalene predominated (ranges: 66–68%) the total PAH analytes in a fuel package comparison study [35]. As the molecular structure of PAH may contribute to the FC of skin wipe samples, the mean of PAH concentration was estimated using the number of fused aromatic rings (Table 3). The range of FC was wide (0.03–2.61) and FC was >1.0 in only four aromatic rings, although FC was statistically significant for the neck. Overall, the results suggest that the number of rings is less likely to be statistically significant, having an inconsistent effect on FC directionality (*n* = 6 with FC < 1.0, *n* = 6 with FC > 1.0).

### 3.4. Meta-Regression of Personal Air Samples

For the personal air samples, the mean of PAH was estimated by the sampling time period (Table 4). After taking into account the number of records needed to maximize statistical power, the sampling time was grouped into shorter (<30 min) and longer (30–60 min) time periods. The mean of PAH concentrations in the shorter time periods ranged from 0.58 to 3.83 µg/m^3^, while the mean in the longer time periods ranged from 23.58 to 45.5 µg/m^3^ (SE of their differences: 0.55–3.86), resulting in an up to 40 times difference. The meta-regression confirmed statistically significant increases in PAH concentrations for longer durations across all IRAC classifications (*p*-values: <0.0001). Similarly, the molecular structure was integrated to understand the impact of sampling period (Table 5). The mean of PAH concentrations in the shorter periods ranged from 1.26 to 18.14 µg/m^3^, while the mean in the longer periods ranged from 24.97 to 223.0 µg/m^3^ (SE of their differences: 0.50–12.62), resulting in an up to 20 times difference. The meta-regression confirmed statistically significant increases in PAH concentrations for longer periods across all molecular structures (*p*-values: <0.0001). Regardless of sampling time, the lower the number of fused aromatic rings, the higher the mean of PAH concentrations.

## 4. Discussion

### 4.1. PAH Exposure Routes—Inhalation and Dermal

Some airborne contaminants can penetrate or permeate turnout gear or between the fabric seams and then be absorbed dermally [45]. Contributing risk factors may result from exposure profiles (e.g., duration, frequency, and proximity) or different adsorption rates of turnout gear materials [19,23]. As previous studies have pointed out, dermal exposure from turnout gear is more of a contributing factor for cancer than inhalation due to three reasons. First, the three layers of material in turnout gear (outer shell, moisture barrier, and thermal liner) are fire-resistant [52] but are not chemical-resistant. Therefore, firefighters may not be protected from PAH, which deposit on the layers and may off-gas. Second, the work environment of firefighters differs considerably from that of other occupational groups. The severe thermal conditions and moist environment during fire extinguishment, in conjunction with the up to 35 kg of multi-layered turnout gear worn by firefighters, leads to more sweat and a higher core temperature, which may increase dermal absorption rates and alter skin microbiome patterns. Third, during fire suppression, firefighters are required to wear a self-contained breathing apparatus (SCBA), which has the highest assigned protection factor (APF = 10,000). An APF of 10,000 indicates that the SCBA is expected to reduce the concentration of contaminants inside of a respirator to 10,000 times less than that of the outside air and, thus, reduces the risk of inhalation exposure. In particular, studies have characterized the effectiveness of SCBA and SCBA practice by fire stage (i.e., knockdown vs. overhaul). With respect to the efficiency of SCBA, particle concentrations were measured with and without SCBA. A comparison of the two measurements showed that the SCBA provided full protection [53]. In addition, urinary samples of firefighters wearing SCBA were collected during different fire stages [54]. Both spirometry and serum pneumoproteins showed that lung function may decrease in the absence of a SCBA or air-purifying respirator during the overhaul process [55]. More recent studies have campaigned for firefighters to wear SCBA during overhaul because they tend to take the SCBA off as soon as knockdown is completed [20,21].

The amount of time that firefighters were in contact with the contaminated gear was not provided in the reviewed studies. This omission is not surprising as most studies that collect air samples assess inhalation exposure routes. Specifically, time is available for inhalation exposure routes as the sampling time, in conjunction with flow rate, is used to calculate the air volume. As opposed to inhalation, sample location is the main driving factor for assessing dermal exposure routes. Our assumption is that the time period of dermal absorption may be longer than that of direct inhalation because firefighters are likely to wear their turnout gear longer than they are actually engaged in fire suppression. When they are running to the fire scene and returning back to the fire station, they are likely wearing their gear. To the best of our knowledge, no studies have systematically reported the rate of absorption of different PAHs by the firefighters’ body or the speed of penetration through protective clothing—turnout gear. The differences between the dermal and inhalation exposure routes are not crystal clear with respect to the cancer risk, although we assume that parts of the body closer to certain cancer sites are more likely to be relevant.

### 4.2. Physicochemical Characteristics of PAH

Along with the carcinogenic characteristics, we integrated the physicochemical characteristics of PAH. PAH analytes with two or three aromatic rings have low molecular weight (MW) and thus are more volatile and highly hydrophilic. Furthermore, reported values are likely to be underestimated due to loss during transport or storage prior to analysis. PAH analytes with four or more rings have high MW and thus are less volatile, have higher hydrophobicity, and are comprised of solid-phase particulates. In addition, removing or washing PAH with high MW from gear is ineffective [19,33,34,37,38,41]. In a study of PAH deposited on gear after fire activities [19,41], all or significant quantities of the PAH compounds had low MW, including naphthalene. However, the relative concentration by mass of naphthalene was higher than that of the other analytes, implying that PAH with higher MW may be more attenuated [41]. In contrast, PAH with higher MW was primarily found on the gear, indicating particle-phase PAH. Thus, gas-phase PAH on gear is less likely to be a pathway for exposure [23].

### 4.3. Exposure Dose

We were not able to examine the correlation between sampling time and concentration of PAH in the meta-analysis. Instead, we verified that the duration of time spent in the fire is the key risk factor for a high dose. Staying the shortest length of time possible in a fire is important, even in live training. For a complete calculation of the dose–response relationship, the exposure matrix needs better estimates based on an exposure assessment. Yet, it is particularly challenging to assess exposure levels for firefighters because they work in random and intermittent fire suppression activities, performing multiple tasks; are often only part-time workers (e.g., volunteer firefighters); and are exposed to intensive fire smoke for an unpredictable duration. Furthermore, note that PAH are not the only candidates among the numerous fire smoke contaminants that can potentially cause cancer [18].

### 4.4. Variability and Homogeneity

Firefighters comprise a unique cohort: they work in random locations for uncertain durations without any advance notice. Even if samples from the original studies are carefully pooled for a meta-regression analysis, thereby allowing for a certain level of comparability, it is extremely difficult to compare study results due to differences in exogenous (e.g., wind) and endogenous (e.g., fuel materials and structural constructions) factors in various fire settings. Bearing this complication in mind, we anticipated highly heterogenous exposure levels in the meta-analysis. Other potential explanations for variations in air samples could be different types of measurement techniques, durations of measurement, air samplers, or analytical methods. In a similar vein, variations in the use of wipe samples were apparent. The original studies selected different skin sites to wipe or to attach fabric swatches to the gear. In addition, although training institutes and fire departments have standard operating procedures and/or uniform requirements [34], an individual firefighter may have different turnout gear or follow other PPE practices for maintenance, usage, and storage.

There are many other confounding exogenous variables in a fire ground (both controlled and emergency fire suppression). Seasonal changes in temperature and wind may affect the behavior of firefighters [37]. During winter, firefighters tend to stay inside the firehouse for controlled fires such as live fire training and vice versa. A firehouse may be located by a fire engine or a shipping container; thus, firefighters may be exposed to different levels of PAH when they stand by. Finally, exposure levels in a fire ground are more likely to be high when firefighters are downwind of a burning structure [35]. For emergency fire responses, sampling variabilities may fluctuate more widely. Due to the logistical issues of an emergency fire, collecting environmental exposure samples may be challenging for a field researcher. Thus, each firefighter may collect his or her own samples using a toolbox with sampling instruments and kits, which may lead to sampling errors. Typically, an exposure assessment is performed by a researcher to minimize any errors. Additional sampling errors may occur due to the uncontrolled nature of a field study, unlike the controlled environment in a laboratory setting. Each research group in the original studies utilized different equipment for sampling and different analytical procedures. In particular, there were significant geographical variations [33]. The studies in North America tended to follow the standard analytical methods established by the EPA and the NIOSH and the restricted guidelines for live fire training evolutions [56]. By contrast, the studies in Europe followed the reference method used by the particular analytical laboratory. To the best of our knowledge, no standard guidelines exist. Furthermore, training environments may not present the same work rate and physiological response [41]. For instance, a one- or two-story training structure is relatively smaller and contains fewer obstacles than those found at an emergency fire. Additionally, more personnel and unexpected task assignment changes may be needed at an emergency fire [34]; thus, the work may be more intense.

### 4.5. Live Fire Training and Emergency Fires

Live fire training and emergency fires differ depending on the fuel materials. In both live training and an emergency fire, the conditions of the burn produce a unique pattern, thus determining the magnitude of exposure [57]. According to the NFPA 1403 Standard for Live Fire Training Evolutions [56], wood products are acceptable as fuel for most training, although furniture is sometimes used to mimic a situation in a municipal fire [34]. Examples of wood products are firewood, particle chipboard, and plywood. However, fuel materials treated with synthetic adhesives may contain specific chemicals that are contaminated with chlorinated or fluorinated compounds [35]. Particle board planks are known to emit more PAH than other materials that do not use glue [33,43]. In the comparison reported in the fuel package study [35], all but one exercise produced lower levels of PAH in live training (range: 2.78–14.2 mg/m^3^) as compared to emergency fires (range: 17.8–23.8 mg/m^3^). The exception in the exercise, an exercise similar to the attack (23.8 mg/m^3^) and search (17.8 mg/m^3^) tactics conducted in a residential fire study [45], used engineered wood products (median = 34.0 mg/m^3^). This finding implies that using engineered wood products for training exercises releases more toxins than untreated materials such as pallets and straw. Other fuel material comparison studies [43,58] recommend the use of safe materials such as wood without glue or gas with 80% ethanol firing liquid. Interestingly, one study [37] found that only wood pallets generated higher total PAH and pyrene concentrations on the skin as well as 1-OHP biomarkers in the urine than mixed fuel (wood pallet with electrical cords and mattresses) in a firefighting exercise. Smoke exposure in live fire training depends on not only the type but also the quantity of fuel materials burned. Typically, fuel materials in live fire training are less likely to be completely consumed [35]. Other contributing factors are the arrangement of fuel and compartment characteristic such as size, layout, ventilation conditions [23,41], fire temperature, burning techniques (wet vs. dry), and ambient conditions during the training exercises [40].

Emergency fires are less likely to have the same conditions from one to another, yet personal air PAH exposure levels in emergency fires are higher than in live fire training. One possible reason is that the fuels burned in emergency fires vary widely from residential materials (e.g., flooring, upholstery, paint, bedding, draperies) to industrial materials (e.g., petroleum, gasoline, combustible metals). A second possible reason is that emergency fires typically evolve for longer, grow hotter, and then become oxygen-limited, whereas training fires are typically fuel-limited. In this review, most environmental PAH exposure assessments were performed under controlled fire conditions, such as live fire training or simulation exercises, rather than in uncontrolled emergency fires. Since 2000, only two studies focused on emergency fires. Assessing exposure levels at an uncontrolled emergency fire is extremely challenging due to the lack of foreknowledge regarding not only the time and location but also the structural type of fire and the fire behavior. Typically, field-based exposure assessments are coordinated well in advance and the participants are shadowed. While shadowing may allow for observational notes, it is not feasible to enter a burning structure. To overcome these unique circumstances, a simulation of the field work will be conducted before the main study. The simulation, which will cover all actions from receiving a call from the dispatcher to transporting the environmental and biospecimen samples to the laboratory, will be more constructive for assessing the logistics. Then, a follow-up discussion with the fire chief, dispatcher, research team, physicians, and medical teams, if applicable, may be arranged to further improve the logistics of the study. Particularly with respect to air sampling, collection is far more challenging than for a training fire. The sampling pump may not run for the entire fire suppression from the beginning of the attack to the end of the search. Due to the vigorous and intense physical activity, a sampling pump attached to a firefighter’s breathing zone is easily detached. For practical purposes [36], one study recommended the use of a passive sampling method as an alternative to active sampling. Unlike active sampling, passive sampling does not require a pump or flow calibrator before and after sampling, which may fit better when a firefighter runs into an emergency fire.

The work processes and task/role assignments in a fire suppression may affect firefighters’ exposure to PAH. Ambient airborne PAH level for overhaul, search, and attack tasks is much higher than for command/pump tasks or outside ventilation [34,45]. For the overhaul task, firefighters enter a smoke-filled structure after fire suppression to search for any possible flames or smoldering materials [42]. Similar to overhaul, search and rescue firefighters are exposed to higher levels of contaminants than fire extinguish firefighters because the latter are able to deflect the smoke, thereby reducing their chemical exposure [40]. By contrast, an incident commander or pump operator is stationed outside the structure and thus is likely to be exposed to less smoke [39]. Furthermore, as they tend not to enter the structure, they rarely wear respiratory protection such as a SCBA [45]. Overall, live training fire studies may overestimate (e.g., overhaul, search, attack) or underestimate (e.g., command/pump) the PAH exposures associated with emergency fire suppression activities [34]. One exception is that instructors at live fire training in the U.S. are likely exposed to higher levels of PAH as multiple training exercises are conducted on a single day, whereas trainees are only involved in one of those exercises [18]. Furthermore, some states in the U.S. impose a mandatory requirement that trainees attend live fire training annually. Other U.S. states do not have a similar requirement, while other countries may require more frequent live fire training sessions. The increased time or frequency of exposure as a function of the job is assumed to be linked with cancer [35]. This exception may only apply to the U.S. One difference is that other countries require more or less live fire training, but we did not investigate all differences from country to country and there may be other differences we are not aware of. As exposure to smoke at a fire scene is heavy in both live fire training and emergency fires, firefighters should conduct gross contamination at a fire scene [27,59]. We also recommend temporarily storing contaminated gear in an airtight container for transportation between the fire scene and the fire department, and then immediately washing and hanging it in a well-ventilated locker or storage area so it can dry. Alternatively, all contaminated turnout gear from firefighters at the fire scene can be directly delivered to the independent service providers for advanced gear cleaning [60].

## 5. Conclusions

Our meta-regression analysis quantified a magnitude of PAH exposure levels for dermal and inhalation routes in the working environment of structural firefighters. The analysis confirmed that they are at a high risk of exposure to PAH when firefighting. We identified various risk factors that contribute to estimates of the effects of exposure at fire activities. The findings indicated that more systematic approaches to methodological and standardized occupational exposure assessments, data analysis, and reporting should be taken. Although assessing exposure levels in an uncontrolled emergency fire is extremely challenging, more research is needed into emergency fires, not only of PAH but also of newer chemical classes of fire smoke retardants and occupational exposure pathways. Evidence-based PAH exposure assessments are critical for determining exposure-dose relationships in large epidemiological studies of occupational risk factors.

## Figures and Tables

**Table 1:** Descriptive characteristics of the selected articles on environmental exposure to polycyclic aromatic hydrocarbons (PAH).

Study ID	Author (Year)	Country/ State (City)	Type of Fire Activity	Study Design	Analytical Method	Analytical Equipment	Sample Type	Number of Participants	Number of Burns	Other Co-Environmental Exposures Measured	Fuel Material
1	Abrard (2019) [33]	France/Beaucouzé	Live fire training	Pre and post fire (+after laundering)	N/A	HPLC	Surface (cloth patch for gear and wipe for helmet)	N/A	1–12 training session(s)	Only BaP as a marker to relate PAHs	Particle board plank, wooden pallet
4	Keir (2020) [34]	Canada/Ottawa	Emergency fire suppression	Pre and post fire (+after laundering)	EPA 3640A	GC/MS	Air (personal, 2.5 lpm; area monitoring at fire station), wipe (skin, gear, and PPE)	Recruited 28 but only 16 participated from 4 fire stations	18 fire suppression events	Metals (antimony, cadmium, and lead)	Involved structural components of residential or commercial buildings
9	Fent (2019) [35]	US/Illinois	Live fire training	Repeated-measures design	NIOSH 5528	N/A	Air (personal, 1 lpm; area), but only considered personal because of no PAH in area	24 firefighters (22 M/2 F) + 10 instructor (9 M/1 F)	3 scenarios based on 3 fuel types	Volatile organic compounds (VOCs), hydrogen cyanide (HCN), aldehydes, acid gases, isocyanates	1) Pallet (pinewood) and straw, 2) Oriented strand board, 3) Simulated smoke
13	Sjostrom (2019) [36]	Sweden/Stockholm	Live fire training and Emergency fire suppression	During and post fire	N/A	GC/MS	Air (active, 2 lpm; passive in training and in emergency), dermal (tape stripping)	7 trainers and 8 team leaders	7 training sessions, 8 emergency fire events	Total dust, VOCs (benzene, 1,3-butadiene)	Typical furnished house with mostly pallets for training; houses, flats, and car materials for emergency live fire
19	Andersen (2018) [37]	Denmark/Copenhagen	Live fire training	Cross-over	EPA TO-13A	GC/MS	Wipe (skin), biomarker	53 trainees (41 male, 12 female)	4 campaigns	PAH only	Wood pellet or mixed (wood pellet + electrical cords and mattresses)
23	Stec (2018) [38]	UK/England	Live fire training	Pre and post fire	N/A	GC/MS	Wipe (skin, PPE, and work surface at fire station)	4 (3 trainees and 1 instructor)	1 training exercise	PAH only	Oriented strand board
25	Wingfors (2018) [18]	Sweden/Sandö	Live fire training	Pre and post fire	N/A	GC/MS	Air (personal, 2.5 lpm), wipe (skin), biomarker	20	3 exercises	PAH only	Lighter fluid, wood wool, untreated chipboard wood
32	Fent (2017) [39]	US/Illinois	Controlled residential fire responses	Pre and post fire	NIOSH 5506	HPLC/UV/FL	Wipe (skin, PPE)	40 (36 M/4 F)	12 scenarios (1/day and no more than 4 scenarios/person)	VOCs (BTEX, styrene), HCN	Fully furnished with mid-20th century structured/dry wall
39	Fernando (2016) [40]	Canada/Ontario	Live fire training	Pre and post fire	N/A	GC/MS	Air (personal, 2 lpm), wipe (skin), biomarker	28 (24 M/4 F)	4 burns	Methoxyphenol (MP)	Untreated wood (e.g., pine, oak, straw)
44	Kirk (2015a) [41]	Australia/Brisbane	Live fire training	Pre and post fire (+ after laundering)	EPA TO-13A	GC/MS	Wipe (PPE) using fabric swatches attached to gear (air samples were excluded due to off-gassing of PAH from post-fire within bag)	N/A	4 evolutions on each of 3 separate days	PAH, VOCs, Carbonyl compounds	Particle board (resin-bonded wood panel)
45	Kirk (2015b) [19]	Australia/Brisbane	Live fire training	Pre and post fire	EPA TO-13A	GC/MS	Air (personal, 2 lpm), wipe (PPE) using fabric swatches attached to gear	N/A	5 evolutions	PAH only	Particle board (resin-bonded wood panel)
48	Baxter (2014) [42]	US/Ohio	Live overhaul event	Comparison of live fire with controlled site	NIOSH 5515	GC/MS	Air (personal, 2 lpm; area), wipe (skin)	10	2 fire stations, 5 live fire, office	PM2.5, Particle (0.02-1µm)	Not specific; various materials in random live overhaul fire
53	Fent (2014) [23]	US/Illinois	Live fire training	Pre and post fire	NIOSH 5506	HPLC	Air (personal, 2.5 lpm), wipe (skin), biomarker	15 (12 repeated)	3 burns × 2 rounds	PAH only	Drywall with typical family room furniture
59	Laitinen (2012) [43]	Finland/Kuopio	Live fire training	Comparison of burn materials	N/A	HPLC	Air (stationary, area), wipe (skin), biomarker	13 (11 conventional + 2 modern simulators)	3 burns per day × (3 days [conventional] + 1 day [modern])	VOCs	Conventional (conifer plywood, chipboard, pine, spruce wood); Modern (propane)
90	Beitel (2020) [44]	US/Arizona	Controlled fire	Pre and post fire	N/A	PAH CALUX	Wipe (skin), biomarker	11	1 controlled burn	PAH only	Wood/w a pallet base, a padded armchair, particle board shelving
120	Fent (2018) [45]	US/Illionis	Controlled residential fire responses	Repeated-measures design	NIOSH 5506	HPLC/UV/FL	Air (personal, 1 lpm; area)	40 (36 M/4 F)	>12 scenarios (fire attack, search, overhaul [backup and RIT, rapid intervention team], outside ventilation, command/pump)	VOC, HCN, PM, Acid gases	Full household furnishings (common in 21st century single family)
150	Hunter (2014) [46]	Sweden/Umea	Live simulator	Cross-over	N/A	HPLC-GC/MS	Air (area)	16	2 occasions	PM1, carbon monoxide, nitrogen oxides, EC/TC (elemental carbon/total)	Wood smoke
257	Ruokojarvi (2000) [47]	Finland/Kuopio	Live fire training	Comparison based on scenario	N/A	GC/MS	Air (unspecified flow rate), wipe (surface)	N/A	N/A	PCB, CPhs, PCDD/Fs	Chipboards, old furniture
258	Bolstad-Johnson (2000) [48]	US/Arizona	Emergency fire suppression	Repeated-measures design	NIOSH 5515	GC	Air (personal, 2 lpm; other components using personal and area)	12 FF	25 fire scenes (14 houses, 6 apartments, 5 commercial b/d)	Asbestos, Cd, Cr, Pb, total dust, Acetaldehyde, Acrolein, Benzaldehyde, BTEX, Formaldehyde, Glutaraldehyde, HCN	Varies from commercial to residential contents
264	Feunekes (1997) [49]	Netherlands/Den Helder	Live fire training	Pre and post fire	Dutch standard NVN 2861	HPLC	Air (personal, 2.1 lpm)	47 (all M)	4 fire exercises	PAH only	N/A (only mentioned “ignited by gasoline”)

**Table 2:** Pre/post comparison of PAH collected from wipe-on-skin samples by IARC group (unit: ng/cm^2^). Bold *p*-value indicates statistically significant dermal exposure increased after the fire activity.

PAH Analyte by IARC Classification ^a^	Skin Sample Location ^b^	No. Records Combined	Overall		Post-Pre Fire Activity		Fold Change (post/pre)	*p*-Value
			Mean	SE	Mean	SE		
Group 1	All	12	0.14	0.03	−0.13	0.10	0.51	0.220
	Neck	3	1.64	0.95	−0.19	2.74	0.89	0.947
	Wrist	4	0.17	0.08	0.1	0.25	2.0	0.681
Group 2B	All	78	0.28	0.01	−0.25	0.03	0.46	<0.001
	Collarbones	14	0.06	0.01	0.02	0.01	1.4	0.157
	Neck	24	1.26	0.08	−0.46	0.14	0.69	0.001
	Wrist	21	0.17	0.02	0.07	0.05	1.70	0.075
Group 3	All	125	0.53	0.01	−0.19	0.03	0.71	<0.001
	Collarbones	13	0.32	0.03	0.1	0.09	1.35	0.258
	Neck	68	1.34	0.05	0.51	0.1	1.43	<0.001
	Wrist	22	0.31	0.03	−0.28	0.08	0.49	<0.001

^a^ Group 1 (*n* = 1): benzo(a)pyrene; no Group 2A due to limited data points for analysis.; Group 2B (*n* = 7): naphthalene, benzo(a)anthracene, chrysene, benzo(b)fluoranthene, benzo(k)fluoranthene, indeno(1,2,3-cd)pyrene, benzo(j)fluoranthene; Group 3 (*n* = 11): acenaphthene, fluorene, phenanthrene, anthracene, fluoranthene, pyrene, perylene, benzo(g,h,i)perylene, 1-methylphenanthrene, benzo(a)fluoranthene, benzo(e)pyrene. ^b^ Limited data points available for analysis at the collarbones in Group 1.

**Table 3:** Pre/post comparison of PAH collected from wipe-on-skin samples by structure (unit: ng/cm^2^). Bold *p*-value indicates statistically significant dermal exposure increased after the fire activity.

PAH Analyte by Structure ^a^	Skin Sample Location ^b^	No. Records Combined	Overall		Post-Pre Fire Activity		Fold Change (Post/Pre)	*p*-Value
			Mean	SE	Mean	SE		
2 rings	All	24	0.95	0.10	−0.58	0.25	0.58	0.020
	Neck	12	1.32	0.19	−0.15	0.46	0.89	0.736
3 rings	All	81	0.47	0.03	0.03	0.06	1.07	0.560
	Collarbones	13	0.26	0.05	−0.41	0.10	0.25	<0.001
	Neck	44	0.48	0.04	0.4	0.09	2.10	<0.001
	Wrist	18	0.43	0.07	−0.26	0.16	0.55	0.101
4 rings	All	81	0.55	0.01	−0.44	0.03	0.49	<0.001
	Collarbones	12	0.13	0.02	0.06	0.04	1.59	0.128
	Neck	44	3.32	0.13	0.68	0.30	1.22	0.024
	Wrist	18	0.21	0.04	−0.06	0.10	1.21	0.695
5 rings	All	53	0.08	0.01	0.08	−0.01	0.89	0.720
	Collarbones	6	0.05	0.004	−0.01	0.01	0.94	0.644
	Neck	9	0.05	0.01	−1.73	0.29	0.03	<0.001
	Wrist	17	0.11	0.02	0.03	0.05	1.23	0.661
6 rings	All	12	0.10	0.01	0.12	0.04	1.50	0.240
	Collarbones	6	0.03	0.01	-0.01	0.02	0.77	0.553
	Wrist	6	0.23	0.07	0.19	0.10	2.61	0.031

^a^ 2 rings (*n* = 3): naphthalene, 2-methylnaphthalene, 1-methylnaphthalene; 3 rings (*n* = 10): acenaphthylene, acenaphthene, fluorene, phenanthrene, anthracene, 3-methylphenanthrene, 2-phenylnaphthalene, 2-methylphenanthrene, 1-methylphenanthrene, 1-methylanthracene; 4 rings (*n* = 5): fluoranthene, pyrene, benzo(a)anthracene, chrysene, 1-methylpyrene; 5 rings (*n* = 7): benzo(b)fluoranthene, benzo(k)fluoranthene, perylene, benzo(a)pyrene, benzo(j)fluoranthene, benzo(a)fluoranthene, benzo(e)pyrene; 6 rings (*n* = 2): Indeno(1,2,3-cd)pyrene, benzo(g,h,i)perylene. ^b^ Limited data points available for analysis at the collarbones in Group 1.

**Table 4:** Collection time and PAH (AM) from air on personal equipment by IARC group (unit: µg/m^3^). Bold *p*-values indicate statistically significant increases in PAH concentrations with time.

PAH Analyte by IARC Classification ^a^	No. Record	Overall		By Time Duration			*p*-Value
		Mean	SE	A: 0–30 Min Mean	B: 30–60 Min Mean	SE of (B-A)	
Group 1	30	7.20	1.05	1.86	33.2	1.88	<0.001
Group 2A	27	6.77	1.00	3.83	45.5	3.86	<0.001
Group 2B	166	7.05	0.55	0.58	23.58	0.55	<0.001
Group 3	247	5.91	0.27	2.92	45.38	1.04	<0.001

^a^ Group 1 (*n* = 1): benzo(a)pyrene; Group 2A (*n* = 2): dibenzo(a,h)anthracene, cyclopenta(c,d)pyrene; Group 2B (*n* = 9): naphthalene, benzo(a)anthracene, chrysene, benzo(b)fluoranthene, benzo(k)fluoranthene, indeno(1,2,3-cd)pyrene, benzo(j)fluoranthene, benzo(b,k)fluoranthene, benzo(c)phenanthrene; Group 3 (*n* = 13): acenaphthene, anthracene, benzo(g,h,i)perylene, fluoranthene, fluorene, phenanthrene, pyrene, 1-methylphenanthrene, 2-methylchrysene, perylene, benzo(e)pyrene, benzo(g,h,i)fluoranthene, benzo(a)fluoranthene.

**Table 5:** Collection time and PAH (AM) from personal air samples by structure groups (unit: µg/m^3^). Bold *p*-values indicate statistically significant increases in PAH concentrations with time.

PAH Analyte by Structure ^a^	No. Records	Overall		By Time Duration			*p*-Value
		Mean	SE	A: 0–30 Min Mean	B: 30–60 Min Mean	SE of (B-A)	
2 rings	86	26.01	2.27	18.14	223.00	12.62	<0.001
3 rings	174	17.38	2.01	11.00	51.84	4.14	<0.001
4 rings	131	9.55	0.73	5.04	43.06	1.96	<0.001
5 rings	141	3.87	0.31	1.26	24.97	0.50	<0.001
6 rings	44	23.97	4.74	. ^b^	.	.	.

^a^ 2 rings (*n* = 7): naphthalene, 2-methylnaphthalene, biphenyl, 1-methylnaphthalene, 2,3-dimethylnaphthalene, 2,3,5-trimethylnaphthalene, 2,6-dimethylnaphthalene; 3 rings (*n* = 12): acenaphthene, acenaphthylene, anthracene, fluorene, phenanthrene, 1-methylflourene, 2-methylphenanthrene, 3-methylphenanthrene, 1-methylphenanthrene, 1-methylanthracene, 2-phenylnaphthalene, retene; 4 rings (*n* = 8): benzo(a)anthracene, chrysene, fluoranthene, pyrene, 1-methylfluoranthene, 1-methylpyrene, 2-methylchrysene, benzo(c)phenanthrene; 5 rings (*n* = 11): benzo(a)pyrene, benzo(b)fluoranthene, benzo(k)fluoranthene, dibenzo(a,h)anthracene, perylene, benzo(e)pyrene, benzo(g,h,i)fluoranthene, cyclopenta(c,d)pyrene, benzo(j)fluoranthene, benzo(a)fluoranthene, benzofluoranthene; 6 rings (*n* = 2): indeno(1,2,3-cd)pyrene, benzo(g,h,i)perylene. ^b^ Limited data points available for analysis.

## Data Availability

The data presented in this study are available on request from the corresponding author.

## Supplementary Materials

The following are available online at https://www.mdpi.com/article/10.3390/ijerph18084209/s1, Supplementary File 1: Search strategies using terms and special features in four databases and Supplementary File 2: Calculation of conversion factor (µg/wipe to ng/cm^2^) for wipe samples [61].
