# Supplementary material for: Health Risks of Structural Firefighters from Exposure to Polycyclic Aromatic Hydrocarbons: A Systematic Review and Meta-Analysis
Source: Int J Environ Res Public Health. 2021 Apr 15;18(8):4209. doi: 10.3390/ijerph18084209 (PMC8071552; doi:10.3390/ijerph18084209)
Supplement: Supplementary file 1 [file ijerph-18-04209-s001.zip › ijerph-1170793-supplementary.pdf]

Supplementary material 2 Calculation of conversion factor (μg/wipe to ng/cm²) for wipe samples

One study collected wipe samples as mass (μg) from the face and neck [42]. For consistency, we converted that unit to ng/m^2. Specifically, the surface area of the human face may be assumed to be 1/3 that of the head [61]. The surface area of the head for an adult male over 21 is 0.154 m^2 and for an adult female over 21 is 0.121 m^2. Thus, given an average head area of 0.138 m^2, we used an area of 0.046 m^2 for the face to calculate the wipe sample in $\mu g/m^2$. Since the surface area of the neck is estimated as a trunk (a much broader area), we referred to another study [38], which estimated that the surface area of the human neck is 0.006 m^2. The summation of these two areas (0.046 m^2 + 0.006 m^2 = 0.052 m^2) was used to represent the skin. If the skin was wiped using a 47mm glass fiber filter (GFF), a conversion factor was implemented as follows: The wipe sample value (ng/wipe) was divided by 17.34cm^2, the surface area of the GFF ($\pi r^2 = \pi$ x $[47mm/2]^2$), resulting in a unit of ng/cm^2 for the meta-analysis. This approach is similar to that used to standardize dermal exposure levels of PAH [39] for the hands and neck.
